# Clinical Risk Factors for Dysphagia and Esophageal Dysmotility in Systemic Sclerosis

**DOI:** 10.3390/jcm12103448

**Published:** 2023-05-13

**Authors:** Mariko Hara, Rumi Ueha, Taku Sato, Takao Goto, Ayumi Yoshizaki, Hayakazu Sumida, Shinichi Sato, Tatsuya Yamasoba

**Affiliations:** 1 Department of Otolaryngology and Head and Neck Surgery, Faculty of Medicine, The University of Tokyo, Tokyo 113-8655, Japan; haram-oto@h.u-tokyo.ac.jp (M.H.); taku.koro.z@gmail.com (T.S.); gottytakao@gmail.com (T.G.); tyamasoba-tky@umin.ac.jp (T.Y.); 2 Swallowing Center, The University of Tokyo Hospital, Tokyo 113-8655, Japan; 3 Department of Dermatology, Faculty of Medicine, The University of Tokyo, Tokyo 113-8655, Japan; ayuyoshi@me.com (A.Y.); sumida-tky@umin.ac.jp (H.S.); satos-der@h.u-tokyo.ac.jp (S.S.); 4 Scleroderma Center, The University of Tokyo Hospital, Tokyo 113-8655, Japan

**Keywords:** systemic sclerosis, esophageal dysmotility, dysphagia, risk factor, autoantibodies

## Abstract

Systemic sclerosis (SSc) is often associated with dysphagia and esophageal dysmotility; however, only a few clinical studies on this topic have been conducted. Patients with SSc who underwent swallowing examinations and esophagography at our institution between 2010 and 2022 were included. A retrospective evaluation of the patients’ backgrounds, autoantibody positivity, swallowing function, and esophageal motility was performed using medical charts. The association between dysphagia and esophageal dysmotility in patients with SSc and respective risk factors was investigated. Data were collected from 50 patients. Anti-topoisomerase I antibodies (ATA) and anti-centromere antibodies (ACA) were detected in 21 (42%) and 11 (22%) patients, respectively. Dysphagia was present in 13 patients (26%), and esophageal dysmotility in 34 patients (68%). ATA-positive patients had a higher risk for dysphagia (*p* = 0.027); ACA-positive patients had a significantly lower risk (*p* = 0.046). Older age and laryngeal sensory deficits were identified as risk factors for dysphagia; however, no risk factors for esophageal dysmotility were identified. No correlation was found between dysphagia and esophageal dysmotility. Esophageal dysmotility is more common in patients with SSc than in those with dysphagia. Autoantibodies can be predictors of dysphagia, and dysphagia must be carefully considered in ATA-positive and elderly patients with SSc.

## 1. Introduction

Systemic sclerosis (SSc) is a chronic multisystem autoimmune disease characterized by tissue fibrosis and vascular abnormalities in the skin, joints, and internal organs such as the esophagus, lower gastrointestinal tract, lungs, heart, and kidneys. The detailed etiology of the disease remains undetermined [1,2,3]. The prevalence of SSc is estimated at 8–30 per 100,000 people in Europe and the United States, with an annual incidence of 1–2 per 100,000 people [1], whereas an incidence of 6,6 per 100,000 people, with a prevalence of 37 per 100,000 people, is found in Japan [2]. SSc is classified as an either diffuse or limited cutaneous disease, based on the extent of skin involvement, and limited systemic sclerosis is more common than diffuse disease [1,2,3]. Anti-topoisomerase I antibodies (ATAs, known as anti-Scl-70), anticentromere antibodies (ACAs), and anti-RNA polymerase III antibodies (ARAs) are the three classic specific antibodies with high validity and reliability [4,5]. Diffuse cutaneous disease (dcSSc) is characterized by positivity for ATAs and ARAs, and limited cutaneous SSs (lcSSc) is characterized by positivity for ACAs [1,6]. dcSSc shows early and rapid organ involvement and poor prognosis, whereas lcSSc shows slower disease progression, visceral involvement later in the disease course, and a better prognosis than diffuse disease [1].

The most common initial symptoms and signs of SSc are Raynaud’s phenomenon, insidious swelling of the distal extremities, and polyarthralgia. Gastrointestinal symptoms or respiratory symptoms are occasionally the first manifestations [1]. Esophageal dysmotility is one of the most frequent complications, present in approximately 90% of patients with SSc, and patients with primarily gastrointestinal symptoms are associated with increased mortality [7,8,9]. Esophageal smooth muscle damage (atrophy and fibrosis), caused by ischemia, nerve damage, and inflammatory factors, can lead to esophageal dysmotility, particularly in the lower esophageal smooth muscle in decreased esophageal clearance [8,9,10]. In addition, dysphagia is the other important comorbidity affecting the quality of life of patients with SSc. Xerostomia, atrophy, and fibrosis of the pharyngeal constrictor muscles can cause oropharyngeal dysphagia [8].

Although the effects of SSc on swallowing function and esophageal motility have been investigated using videofluoroscopy and high-resolution manometry, little has been examined in terms of risk factors for dysphagia and esophageal motility disorders and their association with autoantibodies. Moreover, clinical statistics on esophageal dysmotility and dysphagia in SSc have been reported in Europe and the United States, but these clinical statistics have rarely been reported in Asian regions [8].

In the present study, we first investigated patients with SSc at a tertiary hospital in Japan, with a focus on dysphagia and esophageal dysmotility. Then, we investigated the risk factors for dysphagia and esophageal dysmotility.

## 2. Materials and Methods

### 2.1. Patients and Ethics

We included patients who were diagnosed with SSc based on the 2013 ACR/EULAR classification criteria [3] at the Department of Dermatology, those who presented to the Department of Otolaryngology, the University of Tokyo Hospital, for swallowing evaluation, and those who underwent all swallowing examinations, esophagography, and esophagogastroduodenoscopy (EGD) between 2010 and 2022. The study protocol was approved by the Human Ethics Committee of the University of Tokyo (No. 2487, 2022179NI) and complied with the tenets of the amended Declaration of Helsinki. Written informed consent was obtained from every patient, and patient anonymity was preserved.

### 2.2. Methodology

We conducted a retrospective single-center study using medical charts from our hospital database. We analyzed the clinical and demographic profiles, including age, sex, duration of disease; SSc-related clinical features such as Raynaud’s phenomenon and puffy fingers (thickening and/or swelling of the fingers); presence of autoantibodies; comorbid connective tissue diseases; functional oral intake scale (FOIS) [11] score; oropharyngeal findings; penetration-aspiration scale (PAS) [12] score; esophageal-dilation (ED) score; esophageal endoscopic findings; and medication use such as proton-pump inhibitors (PPIs) and immunosuppressants including steroids (Table S1).

Autoantibodies such as ATAs, ACAs, ARAs, anti-U1 ribo-nucleoprotein antibodies (anti-U1 RNP) associated with the early onset of SSc [13], and other autoantibodies were investigated. The following comorbid connective tissue diseases that met the diagnostic criteria were surveyed: polymyositis/dermatomyositis (PM/DM), Sjogren’s syndrome, systemic lupus erythematosus, rheumatoid arthritis, and antiphospholipid syndrome. To assess oral-intake feeding status, the FOIS score [11] was used; this 7-point ordinal scale reflects the functional diet level of the patient, with higher scores reflecting more normal intake (Table 1). For abnormal oropharyngeal findings, xerostomia was assessed based on the patient’s own complaints, tongue mobility impairment was evaluated by the examiner’s observation, and laryngeal sensory deficit was assessed by the presence or absence of response to touch examinations by the laryngoscope [14].

Dysphagia findings were assessed by videofluoroscopic swallowing study (VFSS) for the following items: velopharyngeal insufficiency, poor laryngeal elevation, reduced pharyngeal contraction, impaired upper-esophageal sphincter (UES) opening, and pharyngeal residue. A PAS score is a widely used means of grading the severity of penetration or aspiration and was evaluated by VFSS and assigned to levels 1–8 (normal level: 1) [15]. The occurrence of penetration-aspiration was defined as PAS scores ≥ 3, a clinically relevant classification for swallowing safety according to previous studies [16,17,18]. In this study, a PAS score ≥ 3 was defined as dysphagia.

**Figure 1:**
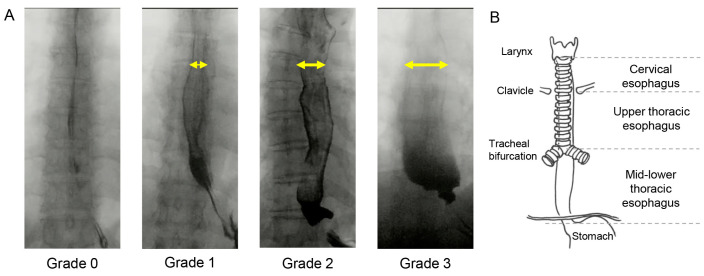
Esophageal-dilation grade according to the esophageal anatomical classification. (**A**): Representative findings for each ED Grade. Yellow arrows indicate dilated esophageal diameter after esophageal contraction. (**B**): Three sections of the esophagus, Yellow arrows indicate dilated esophageal diameter after esophageal contraction.

Esophageal motility was evaluated by videofluoroscopic esophagram (VFE). Two independent investigators quantified the degree of esophageal dilatation as an esophageal-dilation (ED) score. In general, the esophagus dilates when a food bolus passes through it and contracts after swallowing, but in some patients with SSc, the esophagus may remain dilated even after finishing swallowing. The esophageal diameter after swallowing was compared to the esophageal diameter during swallowing. The comparative value was calculated by dividing the diameter of the esophagus after esophageal contraction by the maximally dilated esophageal diameter, and an ED grade of 0 was defined as a value of 20% or less, grade 1 as 20–50% value, grade 2 as 50–80% value, and grade 3 as value of 80% or more (Figure 1A). The esophagus, as visualized fluoroscopically, was anatomically divided into three sections based on a revised classification of previous reports [19,20]: (1) cervical esophagus: proximal to the clavicles, (2) upper thoracic esophagus: from the clavicles distal to the tracheal bifurcation, and (3) mid-lower thoracic esophagus: from the tracheal bifurcation to the lower esophageal sphincter. ED grade was evaluated at these locations, and the ED score was defined as the sum of the ED grades at the three sites (Figure 1B). Herein, an ED score ≥ 3 was defined as having apparent esophageal dysmotility, since even healthy elderly patients without esophageal symptoms have mild esophageal dysmotility (ED score 1–2) [20]. The PAS and ED scores were evaluated based on a consensus between two otolaryngologists with at least 10 years of experience. In case of disagreement between the two evaluators, the rating was determined from the recordings and mutual discussions.

Then, we classified all patients into two groups, those with dysphagia (PAS ≥ 3) and those without dysphagia (PAS score ≤ 2) upon swallowing thin liquids, and the clinical risk factors for dysphagia in the patients with SSc were examined. Furthermore, to investigate clinical factors contributing to esophageal dysmotility in patients with SSc, we divided all patients into two groups based on the ED scores (those with ED score ≤ 2, those with ED score ≥ 3), and the differences in clinical background between the two groups were assessed. Finally, we examined whether there was a correlation between dysphagia and esophageal dysmotility.

### 2.3. Videofluorographic Study

Videofluorographic studies were performed and recorded in the lateral and anteroposterior views. Iohexol (Omnipaque^®^, Daiichi-Sankyo, Tokyo, Japan) was used as a contrast agent in VFSS [18,20]. First, 3 swallows of 5 mL of thin contrast agent (10 mPa·s) were administered in the lateral view, and then swallowing functions and PAS score were evaluated. Then, the VFE was performed in the anteroposterior view with 3 swallows of 5 mL of thin contrast agent [19,20]. On the second and third examinations, swallows were inducted after clearing the previous intraesophageal stasis. ED scores for each esophageal site were evaluated.

### 2.4. Statistical Analyses

We analyzed all data using BellCurve for Excel (version 4.03; Social Survey Research Information Co., Ltd., Tokyo, Japan) and examined the associations among clinical and demographic profiles for penetration-aspiration and esophageal dilation. We used Mann–Whitney’s test to analyze continuous variables and the chi-square test or Fisher’s exact test to analyze categorical variables. Odds ratios (ORs) were calculated for each event that occurred and for items for which a 2 × 2 cross-statistics table could be generated. *p* < 0.05 was considered statistically significant. Spearman’s rank correlation coefficient was computed to assess the relationship between penetration-aspiration and esophageal dilation.

## 3. Results

### 3.1. Patient Demographics

**Figure 2:**
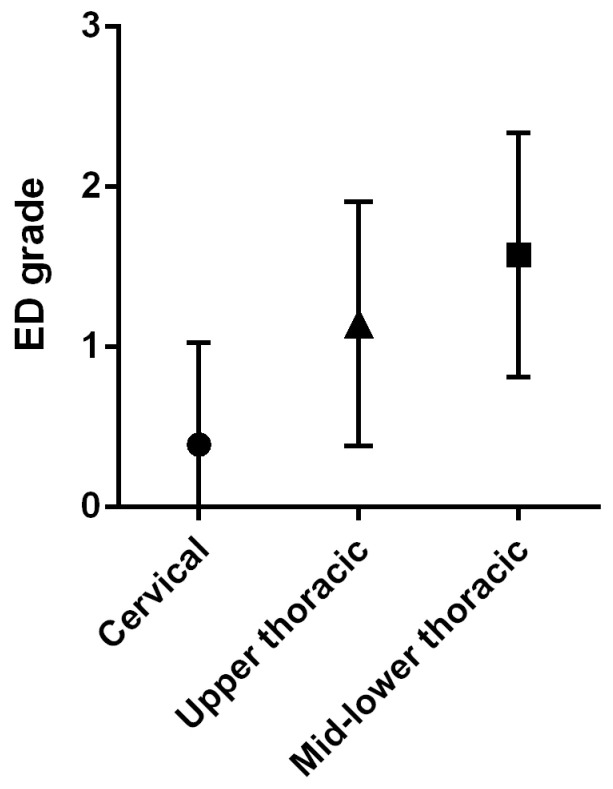
Esophageal-dilation grade by esophageal site. ED: esophageal dilation.

Table 1 lists the demographic data of the enrolled patients. We identified 50 eligible patients. The median age at the time of VFSS was 61 years (interquartile range (IQR), 50–69 years), and there was a female predominance (88%). The median duration of disease at baseline was 110 months (IQR, 33–265 months). Of serum autoantibodies related to SSc, ATAs are detected in 21 patients (42%), ACAs in 11 patients (22%), ARAs in 2 patients (4%), anti-U1 RNP antibodies in 9 patients (18%). Almost all patients presented with Raynaud’s phenomenon and/or puffy fingers. The most common comorbid connective tissue disease was PM/DM in 17 patients (34%), followed by Sjogren’s syndrome in 12 patients (24%). The median FOIS score was 7 (IQR, 6–7), and all subjects took it orally at every meal. Xerostomia was present in 14 patients (28%), and laryngeal sensory deficits in 20 patients (40%). Dysphagia findings assessed by VFSS included reduced pharyngeal contraction in 25 patients (50%) and poor laryngeal elevation and pharyngeal residue in 16 patients (32%). The median ED score was 3 (IQR, 2–4), and the score was highest in the mid-lower thoracic esophagus (Figure 2). Esophageal endoscopy revealed GERD in 21 patients (42%). Four patients (8%) did not have esophageal dilatation on videofluorographic study despite the presence of GERD on endoscopy, while 17 patients (34%) had esophageal dilatation despite the absence of GERD on endoscopy. All except two patients had taken PPIs orally, and approximately 80% of patients had been treated with immunosuppressants.

### 3.2. Clinical Risk Factors for Dysphagia in Patients with SSc

Next, to examine the clinical risk factors for dysphagia (PAS score ≥ 3) in patients with SSc, the patients were classified into two groups: those with penetration-aspiration (PAS score ≥ 3) and those without penetration-aspiration (PAS score ≤ 2) upon swallowing thin liquids. The variables listed in Table 2 as clinical factors were evaluated. Patients with serum ATA-positive and serum anti-U1 RNP-antibody-positive were at a higher risk of dysphagia, whereas those with ACA-positive had a lower risk of dysphagia. Older age and laryngeal sensory deficits were significantly associated with dysphagia, while no significant association was observed with sex, duration of disease, and comorbidities. Moreover, there was no significant clinical association between esophageal dysmotility (ED score ≥ 3) and dysphagia.

### 3.3. Clinical Risk Factors for Esophageal Dysmotility in Patients with SSc

To examine the clinical risk factors for esophageal dysmotility in patients with SSc, the patients were classified into two groups (ED score ≤ 2, ED score ≥ 3), and the differences in clinical background between the two groups were assessed. Of the many clinical factors and features, only xerostomia was found to be a risk factor for esophageal dysmotility. Unexpectedly, GERD and hiatal hernia were not significantly associated with the occurrence of esophageal dysmotility. Furthermore, none of the autoantibodies relevant to SSc were found to be associated with an increased risk of esophageal dilation (Table 3). Finally, we examined a correlation between dysphagia (PAS score ≥ 3) and esophageal dysmotility (ED score ≥ 3), but there was no relationship between the two variables (r = 0.19, *p* = 0.20).

## 4. Discussion

The present study demonstrated that patients with SSc, who were positive for ATA, anti-U1 RNP antibodies, were at higher risk for dysphagia and that older age and laryngeal sensory deficits were risk factors for dysphagia. Similarly, this study showed that esophageal dysmotility was present in the majority of patients with SSc and was more frequent than dysphagia. However, risk factors for esophageal dysmotility could not be determined.

### 4.1. Systemic Scleroderma and Dysphagia

It has been reported that approximately 60% of scleroderma patients are aware of dysphagia [21]. The oral phase of swallowing is easily impaired, and reduced salivary flow is a typical symptom [22]. Abnormalities at the pharyngeal phase of swallowing have also been reported in more than half of the patients with SSc, including pharyngeal residue, penetration, and aspiration [22,23]. The possible mechanisms of dysphagia in SSc include irreversible neuropathy due to tissue hypoxia caused by vascular damage and dysfunction of the oropharyngeal muscles due to muscle fibrosis and collagen deposition [21,24]. In addition, overlapping autoimmune syndromes, such as cases of co-existing inflammatory myositis or myasthenia gravis, may contribute to dysphagia by impairing normal pharyngeal muscle function [24]. Moreover, patients with SSc may also experience pharyngeal inflammation related to uncontrolled gastroesophageal reflux disease (GERD) [24]. The present study demonstrated that the following swallowing functions were impaired in the dysphagia (penetration-aspiration) group compared to the non-dysphagia (without penetration-aspiration) group: pharyngeal contraction, pharyngeal retention, laryngeal sensation, laryngeal elevation, and UES opening. It is speculated that pharyngeal swallowing function is impaired due to atrophy and fibrosis of the pharyngeal constrictors and the anterior cervical muscles responsible for swallowing movements. In addition, neuropathy may interfere with the coordinated movements of swallowing, thereby disturbing smooth swallowing movements.

Regarding xerostomia, which is considered to be one of the causes of dysphagia in patients with SSc [21,24], it was not a risk factor for penetration-aspiration in this study. Although it should be noted that this result was evaluated based on subjective symptoms and not objective evaluation data, it is conceivable that xerostomia alone would have little effect on penetrarion-aspiration; instead, other factors during the pharyngeal phase of swallowing may have affected penetrarion-aspiration. Laryngeal sensory deficits have been known to be a risk factor for aspiration pneumonia [14], and this is the first report demonstrating that laryngeal sensory deficits can influence penetration-aspiration in patients with SSc. It was also suggested that neuropathy caused by the blood flow disturbance based on SSc could occur in the laryngeal area. Among the items evaluated for dysphagia, laryngeal elevation, laryngeal penetration, and UES passage are more appropriately assessed by VFSS than by endoscopy. However, laryngeal sensation and vocal fold movement are more appropriate for evaluation by endoscopy than by VFSS. Hence, both endoscopy and VFSS should be performed in patients with SSc, depending on the purpose of the evaluation. Although it has been reported that almost all patients with pharyngeal dysphagia showed signs of altered esophageal clearance or reflux disease [22], the present study found no correlation between penetration-aspiration and esophageal dysmotility.

### 4.2. Autoantibodies in Systemic Scleroderma and Dysphagia

SSc can be classified into dcSSc and lcSSc. In dcSSc, which has a strong systemic effect, autoantibodies ATAs and ARAs are often positive, while in lcSSc, which has a less systemic effect and a slower disease progression than dcSSc, autoantibodies ACAs tend to be positive [1,6]. The present study demonstrated that ATAs-positive patients are at risk for dysphagia, while ACAs-positive patients are at low risk for dysphagia. In other words, in patients with SSc, patients with systemic effects of the disease were more likely to develop dysphagia. The association between the expression of autoantibody ARAs and dysphagia remains unclear. Since the number of subjects in this study was small, further investigation is needed in the future. Regarding anti-U1 RNP, the present study showed that positivity for anti-U1 RNP is associated with the risk of dysphagia. However, since there have been conflicting reports of both well [25] and poor [26] prognosis in anti-U1 RNP-positive patients in previous studies, the clinical course of dysphagia in anti-U1 RNP-positive patients should be carefully monitored in the future.

### 4.3. Systemic Scleroderma and Esophageal Dysmotility

Esophageal disorders are present in 60–90% of patients with SSc in European and North American populations [8,22,24,27,28], and the present study showed that esophageal dysmotility was present in 68% of all cases, which is consistent with the results of these previous reports. The esophagus is involved in the middle and distal tract. Esophageal dysmotility included abnormal motility of UES (13%) and LES (76%), inadequate primary peristalsis (52%), and non-peristaltic contractions (40%) [22]. Altered peristalsis is typically observed in the distal esophagus, but it also involves the proximal third in the advanced phase. The lumen of the esophagus gradually becomes extended [28,29]. This study also identified the most dilated esophagus (ED score 2 or 3) in the mid-lower thoracic esophagus. Esophageal motility disorders can be evaluated by manometry in addition to by VFE. Further, an esophageal manometric study revealed that esophageal motility disorders can develop during the early disease phases of SSc [30].

The severity classification of the upper-gastrointestinal tract lesions, the Japanese SSc clinical guideline defined normal esophageal motility as (grade 0: normal), reduced peristalsis of the lower esophagus (grade 1: mild), gastroesophageal reflux disease (grade 2: moderate), reflux esophagitis with dysphagia (grade 3: severe), and esophageal stenosis-related dysphagia (grade 4: very severe) [31]. We identified approximately 40% of patients in whom the presence of GERD in EGD did not match the presence of esophageal dysmotility in VFE. It has also been reported that 18–40% of patients with SSc may have asymptomatic esophageal dysmotility [32]. This means that a severity diagnosis of SSc based solely on EGD findings may underestimate esophageal motility disorders. Approximately 75–85% of patients with SSc who met the 2013 ACR/EULAR classification criteria [3] had no esophageal lesions on endoscopy but had esophageal motility disorders on high-resolution manometry (HRM) [33,34,35]. This means that EGD alone is not sufficient to evaluate esophageal function. Given that esophageal HRM is more physically invasive than VFE, VFE should be the first choice for esophageal motility evaluations. Although EGD is useful in evaluating esophageal lumen findings, VFE, which allows for the real-time dynamic evaluation of esophageal motility, is considered more appropriate for evaluating esophagus in patients with SSc.

This study had several limitations. The retrospective chart review was limited by incomplete or missing documentation. Multivariate analysis could not be performed to verify the results because of the small sample size. Furthermore, although most of the patients in this study were prescribed PPIs due to medical reasons, the effects of PPIs on esophageal motility cannot be ruled out. This study did not include all the patients with SSc who presented to our institution. Only the patients with swallowing discomfort symptoms, such as pharyngeal residue sensation or chest pain when swallowing, who were referred to the Department of Otolaryngology for the purpose of a swallowing evaluation, were included. In addition, patients who underwent all swallowing examinations, esophagography, and EGD were included in this study, which may have led to a patient selection bias. Moreover, since comorbid PM/DM increases the risk of dysphagia, it is possible that increased attention was paid to the swallowing function in SSc patients with PM/DM and patients with PM/DM were more frequently referred for swallowing function evaluation purposes. This may have increased the comorbidity rate of PM/DM among patients with SSc.

## 5. Conclusions

Esophageal dysmotility was common in SSc and was more frequent than dysphagia. Although this study did not reveal the factors contributing to esophageal dysmotility, autoantibodies can be a predictor of dysphagia. Moreover, dysphagia must be carefully considered in ATAs-positive and elderly patients with SSc.

## Figures and Tables

**Table 1:** Patient demographics.

Characteristic	
Patients, no.	50
Age, years, median (IQR)	61 (50, 69)
Female, no. (%)	44 (88%)
Duration of disease, months, median (IQR)	110 (33, 265)
Autoantibodies, no. (%)	
Anti-topoisomerase I antibodies (ATAs)	21 (42%)
Anti-centromere antibodies (ACAs)	11 (22%)
Anti-RNA polymerase III antibodies (ARAs)	2 (4%)
Anti-U1 RNP antibodies	9 (18%)
Other autoantibodies	11 (22%)
Typical findings of SSc	
Raynaud’s phenomenon, no. (%)	43 (86%)
Puffy fingers, no. (%)	40 (80%)
Comorbid connective tissue diseases, no. (%)	
Polymyositis/Dermatomyositis	17 (34%)
Sjogren’s syndrome	12 (24%)
Systemic lupus erythematosus	6 (12%)
Rheumatoid arthritis	5 (10%)
Antiphospholipid syndrome	4 (8%)
Functional oral intake scale, median (IQR)	7 (6, 7)
Oropharyngeal findings, no. (%)	
Xerostomia	14 (28%)
Tongue mobility impairment	9 (18%)
Laryngeal sensory deficits	20 (40%)
Dysphagia findings, no. (%)	
Velopharyngeal insufficiency	1 (2%)
Poor laryngeal elevation	14 (28%)
Reduced pharyngeal contraction	25 (50%)
Impaired UES opening	11(22%)
Pharyngeal residue	16 (32%)
PAS score, median (IQR)	1 (1, 3)
Esophageal-dilation score, median (IQR)	3 (2, 4)
Esophageal endoscopic findings, no. (%)	
GERD (with/without ED)	4 (8%)/17 (34%)
Non-GERD (with/without ED)	12 (24%)/17 (34%)
Esophageal hiatal hernia	23 (46%)
Medication	
PPIs, no. (%)	48 (96%)
Immunosuppressants, no. (%)	39 (78%)

no.: number, IQR: interquartile range, RNP: anti-ribonucleoprotein antibody, PAS: penetration-aspiration scale, GERD: gastroesophageal reflux disease, ED: esophageal dilation, PPI: proton-pump inhibitor, UES: upper esophageal sphincter.

**Table 2:** Association between clinical factors and penetration-aspiration (PAS score ≥ 3).

	PAS Score ≤ 2	PAS Score ≥ 3	OR (95% CI)	*p* Value
Patients, no. (%)	37 (74%)	13 (26%)		
Age, years, median (IQR)	60 (46, 66)	68 (54, 73)		0.027 *
Female, no. (%)	32 (86%)	12 (92%)	1.88 (0.20–17.74)	1.000
Duration of disease, months, median (IQR)	99 (28, 236)	255 (72, 270)		0.521
Autoantibodies, no. (%)				
Anti-topoisomerase I antibodies (ATAs)	12 (32%)	9 (69%)	4.69 (1.20–18.34)	0.027 *
Anti-centromere antibodies (ACAs)	11 (30%)	0 (0%)	-	0.046 *
Anti-RNA polymerase III antibodies (ARAs)	2 (5.4%)	0 (0%)	-	1.000
Anti-U1 RNP antibodies	4 (11%)	5 (39%)	5.16 (1.12–23.69)	0.040 *
Other autoantibodies	10 (27%)	1 (7.7%)	0.22 (0.026–1.96)	0.248
Comorbid connective tissue diseases, no. (%)				
Polymyositis/Dermatomyositis	11 (30%)	6 (46%)	2.02 (0.55–7.42)	0.322
Sjogren’s syndrome	7 (19%)	5 (38%)	2.68 (0.69–10.73)	0.256
Systemic lupus erythematosus	3 (8.1%)	3 (23%)	3.40 (0.59–19.54)	0.173
Rheumatoid arthritis	4 (11%)	1 (7.7%)	0.69 (0.07–6.78)	1.000
Antiphospholipid syndrome	2 (5.4%)	2 (15%)	3.18 (0.40–25.31)	0.275
Functional oral intake scale, median (IQR)	7 (6, 7)	6 (5, 7)		0.023 *
Oropharyngeal findings, no. (%)				
Xerostomia	11 (30%)	3 (23%)	0.71 (0.16–3.08)	0.734
Tongue mobility impairment	5 (14%)	4 (31%)	2.84 (0.63–12.86)	0.214
Laryngeal sensory deficits	10 (27%)	10 (77%)	9.00 (2.05–39.55)	0.003 **
Dysphagia findings, no. (%)				
Velopharyngeal insufficiency	0 (0%)	1 (7.7%)	-	0.260
Poor laryngeal elevation	4 (11%)	10 (77%)	27.50 (5.25–144.00)	<0.001 ***
Reduced pharyngeal contraction	12 (32%)	13 (100%)	-	<0.001 ***
Impaired UES opening	4 (11%)	7 (54%)	9.63 (2.14–43.36)	0.003 **
Pharyngeal residue	6 (16%)	10 (77%)	17.22 (3.62–81.83)	<0.001 ***
Esophageal-dilation score, median (IQR)	3 (2, 4)	4 (2, 4)		0.397
Immunosuppressants, no. (%)	27 (73%)	12 (92%)	4.44 (0.51–38.74)	0.248

PAS: penetration-aspiration scale, OR: odds ratio, no.: number, IQR: interquartile range, RNP: anti-ribonucleoprotein antibody, no: number, CI: confidence interval. * *p* < 0.05, ** *p* < 0.01, *** *p* < 0.001.

**Table 3:** Association between clinical factors and esophageal dilation (ED score ≥ 3).

	ED Score ≤ 2	ED Score ≥ 3	OR (95% CI)	*p* Value
Patients, no. (%)	16 (32%)	34 (68%)		
Age, years, median (IQR)	52 (44, 66)	62 (53, 70)		0.060
Female, no. (%)	15 (94%)	29 (85%)	0.39 (0.04–3.62)	0.650
Duration of disease, months, median (IQR)	73 (15, 227)	123 (68, 297)		0.167
Autoantibodies, no. (%)				
Anti-topoisomerase I antibodies (ATAs)	7 (44%)	14 (41%)	0.9 (0.27 to 2.99)	1.000
Anti-centromere antibodies (ACAs)	3 (19%)	8 (24%)	1.33 (0.30 to 5.88)	1.000
Anti-RNA polymerase III antibodies (ARAs)	0 (0%)	2 (5.9%)	-	1.000
Anti-U1 RNP antibodies	2 (13%)	7 (21%)	1.81 (0.33 to 9.92)	0.699
Other autoantibodies	3 (19%)	8 (24%)	1.33 (0.30–5.88)	1.009
Comorbid connective tissue diseases, no. (%)				
Polymyositis/Dermatomyositis	6 (16%)	11 (32%)	0.79 (0.23–2.76)	0.757
Sjogren’s syndrome	3 (19%)	9 (26%)	1.56 (0.36–6.77)	0.728
Systemic lupus erythematosus	2 (13%)	4 (12%)	0.93 (0.15–5.71)	1.000
Rheumatoid arthritis	1 (6.2%)	4 (12%)	2.00 (0.21–19.50)	1.000
Antiphospholipid syndrome	1 (6.3%)	3 (8.8%)	1.45 (0.14–15.15)	1.000
Functional oral intake scale, median (IQR)	7 (7, 7)	7 (6, 7)		0.058
Oropharyngeal findings, no. (%)				
Xerostomia	1 (6.3%)	13 (38%)	9.29 (1.09–78.86)	0.020 *
Tongue mobility impairment	1 (6.3%)	8 (24%)	4.62 (0.53–40.58)	0.240
Laryngeal sensory deficits	6 (38%)	14 (41%)	1.16 (0.34–3.96)	1.000
Dysphagia findings, no. (%)				
Velopharyngeal insufficiency	0 (0%)	1 (2.9%)	-	1.000
Poor laryngeal elevation	4 (25%)	10 (29%)	1.25 (0.32–4.83)	1.000
Reduced pharyngeal contraction	7 (44%)	18 (53%)	1.45 (0.44–4.78)	0.762
Impaired UES opening	3 (19%)	8 (24%)	1.33 (0.30–5.88)	1.000
Pharyngeal residue	5 (31%)	11 (32%)	1.05 (0.29–3.78)	1.000
PAS score, median (IQR)	1 (1, 1.5)	1 (1, 2.8)		0.607
Esophageal endoscopic findings, no. (%)				
GERD	4 (25%)	17 (50%)	3.00 (0.80–11.19)	0.129
Esophageal hiatal hernia	5 (31%)	18 (53%)	2.48 (0.71–8.67)	0.225
Immunosuppressants, no. (%)	12 (75%)	27 (79%)	1.29 (0.32–5.24)	0.728

ED score: esophageal-dilation score, OR: odds ratio, no.: number, IQR: interquartile range, RNP: anti-ribonucleoprotein antibody, PAS: penetration-aspiration scale, GERD: gastroesophageal reflux disease, CI: confidence interval. * *p* < 0.05, UES: upper esophageal sphincter.

## Data Availability

The datasets used and analyzed during the current study are available from the corresponding author on reasonable request.

## Supplementary Materials

The following supporting information can be downloaded at: https://www.mdpi.com/article/10.3390/jcm12103448/s1, Table S1: Details of the penetration-aspiration scale and functional oral intake scale scores.
