# Supplementary material for: Clinical Risk Factors for Dysphagia and Esophageal Dysmotility in Systemic Sclerosis
Source: J Clin Med. 2023 May 13;12(10):3448. doi: 10.3390/jcm12103448 (PMC10219285; doi:10.3390/jcm12103448)
Supplement: Supplementary file 1 [file jcm-12-03448-s001.zip › jcm-2371497-supplementary.pdf]

Table S1. Details of the penetration-aspiration scale and functional oral intake scale scores

	Penetration-Aspiration Scale
Score 1	Material does not enter the airway
2	Material enters the airway, remains above the vocal folds, and is ejected from the airway
3	Material enters the airway, remains above the vocal folds, and is not ejected from the airway
4	Material enters the airway, contacts the vocal folds, and is ejected from the airway
5	Material enters the airway, contacts the vocal folds, and is not ejected from the airway
6	Material enters the airway, passes below the vocal folds and is ejected into the larynx or out of the airway
7	Material enters the airway, passes below the vocal folds, and is not ejected from the trachea despite effort
8	Material enters the airway, passes below the vocal folds, and no effort is made to eject

Functional Oral Intake Scale Score	
Level 1	Nothing by mouth
2	Tube dependent with minimal attempts of food or liquid
3	Tube dependent with consistent oral intake of food or liquid
4	Total oral diet of a single consistency
5	Total oral diet of multiple consistencies but requiring special preparation or compensations
6	Total oral diet of multiple consistencies without special preparation but with specific food limitations
7	total oral intake without restrictions
